# The Vision of “Industrie 4.0” in the Making—a Case of Future Told, Tamed, and Traded

**DOI:** 10.1007/s11569-016-0280-3

**Published:** 2017-01-25

**Authors:** Sabine Pfeiffer

**Affiliations:** Department of Sociology, Faculty of Business, Economics and Social Sciences, University of Hohenheim, Wollgrasweg 23, 70599 Stuttgart, Germany

**Keywords:** Visioneering, Industry 4.0, Industrie 4.0, Future, Anticipation

## Abstract

Since industrial trade fair Hannover Messe 2011, the term “Industrie 4.0” has ignited a vision of a new Industrial Revolution and has been inspiring a lively, ongoing debate among the German public about the future of work, and hence society, ever since. The discourse around this vision of the future eventually spread to other countries, with public awareness reaching a temporary peak in 2016 when the World Economic Forum’s meeting in Davos was held with the motto “Mastering the Fourth Industrial Revolution.” How is it possible for a vision originally established by three German engineers to unfold and bear fruit at a global level in such a short period of time? This article begins with a summary of the key ideas that are discussed under the label Industrie 4.0. The main purpose, based on an in-depth discourse analysis, is to debunk the myth about the origin of this powerful vision and to trace the narrative back to the global economic crisis in 2009 and thus to the real actors, central discourse patterns, and hidden intentions of this vision of a new Industrial Revolution. In conclusion, the discourse analysis reveals that this is not a case of visioneering but one of a future told, tamed, and traded.

## The German Debate on Industrie 4.0: a Case of Visioneering?

Since 2011, the notion of “Industrie 4.0” has stimulated an increasingly lively debate in German society. Initially concerned with new technological manufacturing options, the discourse has developed into an ongoing and still-intensifying debate that is reverberating through more and more spheres of society. The future seems to occupy a prominent place on the current discourse agenda, and in drawing on the book *Future Matters* [1], the present article considers whether this future is merely “told” (and by whom) or whether it is also tamed and traded. Although there is professional debate about Industrie 4.0 and the manufactural Internet in other countries too, in Germany the numbers “4.0” have developed into a well-known meme that adorns conferences all over the country, signaling fundamental discussions on no less a topic than the future of work and the future of society as a whole. What McCray [2] terms *visioneering* inevitably comes to mind. The marketing-style term Industrie 4.0 was invented and promoted by three engineers: Henning Kagermann (physicist and one of the founders of SAP), Wolfgang Wahlster (professor of artificial intelligence), and Wolf-Dieter Lukas (physicist and senior official at the German Federal Ministry of Education and Research). What they started in 2011 during a press conference at the Hannover Messe and in 2016 became the main motto of the World Economic Forum (WEF) [3] meeting in Davos almost perfectly matches what McCray defines as visioneering:

“Visioneering certainly embraces a probing and exploratory approach to design as technically fluent experts speculate at to what *can* be built. Yet visioneering also involves the popularization of ideas, the construction of networks of supporters, and the cultivation of patrons” ([2], p. 152).

The latter especially seems to be the ongoing mission of Henning Kagermann, who, as the president of the German National Academy of Science and Engineering (acatech), successfully institutionalized, and continues to maintain, a high-profile network of leading managers, politicians, and other influential figures from employer and business organizations and trade unions to promote the vision of Industrie 4.0. And this notion was framed as a vision right from the outset: the first strategic paper that acatech published on Industrie 4.0 explicitly describes Industrie 4.0 as a “vision” that has to be shaped, and draws a picture of what the “future [will] look like under Industrie 4.0” ([4], p. 18–20). And this future, although driven by technology, magically seems to solve a host of societal problems that were once thought to be insoluble—not only in Germany but across the world:

“In addition, Industrie 4.0 will address and *solve some of the challenges facing the world today* such as resource and energy efficiency, urban production and demographic change. Industrie 4.0 enables continuous *resource productivity and efficiency* gains to be delivered across the entire value network. It allows work to be organized in a way that takes *demographic change* and social factors into account. Smart assistance systems release workers from having to perform routine tasks, enabling them to focus on creative, value-added activities. In view of the impending shortage of skilled workers, this will allow older workers to extend their working lives and remain productive for longer. Flexible work organization will enable workers to combine their work, private lives, and continuing professional development more effectively, promoting a better *work-life balance”* ([4], p. 5; emphasis in original).

However, the strategy paper on Industrie 4.0 is not very specific about how its stated goals, which are in part mutually incompatible, can be achieved. The propagated utopia repeatedly upholds the mantra-like dictum that Industrie 4.0 is completely human-centered and will therefore not pave the way towards deserted, fully automated factories.

With Industrie 4.0, new and ostensibly unheard-of technical possibilities are being touted. Without a doubt, the technical innovations being discussed are fascinating, and the whole enterprise cannot be understood, much less realized, without giving them due attention. Yet, we must always differentiate between the real and speculated effects of technology, between serious discourse and media hype, between true innovation and old hat. A command of basic knowledge about IT systems and production-process technology is often lacking, although it would surely aid objective analysis, as would familiarity with the state of the art of work organization and the intra-logistics of factory assembly. Technical expertise of this sort would quickly demystify much of the current hype.

But even those who coined the term Industrie 4.0 are astonishingly vague about the technical details of the big, visionary picture they paint in the paper cited above. Despite their technical backgrounds, the three engineers seem to deliberately avoid describing technical details linked to concrete applications of Industrie 4.0 on the shop floor and instead indulge in colorful visions of the future. And this is why the vision of Industrie 4.0 lacks one central feature of McCray’s definition of visioneering, which in his words “(…) requires more than imaginative and sweeping ideas for how new technologies might dramatically reshape society. It also requires some application of technical skills, knowledge, and calculations to press forward toward the technological future. Engineering, after all, culminates in building *things*. Successful visioneering demands that paper, pencils, and the speaker’s podium be exchanged for the hammer, forge, and welder’s arch” ([2], p. 261, emphasis in original).

Nevertheless, this article cannot take up the tasks, important as they are, of elaborating on the technical phenomena associated with Industrie 4.0, the technological ontologies that underlie them, or their sociological implications. Even a discussion of technical issues alone would fill more pages than appropriate for a single article. Instead, this article uses the now quite expansive reach and the loud buzz surrounding Industrie 4.0 as a jumping-off point for an in-depth examination of the strategies and actors behind it all.

The buzz surrounding Industrie 4.0 has put the spotlight back on industrial production and industrial employment after it was very nearly relegated to historical texts. For many years and until just recently, industrial production was “old economy,” both for policymakers and academic researchers. Production and assembly work was written off as being in permanent decline, just industrial residue in a post-industrial society. Although knowledge about these work environments was current in society and research as recently as just 20 years ago, it has since faded like old photographs. Thus, I will concentrate in this article on the industrial core and its immediate edges, to the neglect of equally important processes that have initiated new forays into digitalization such as crowd sourcing, micro work, and the emerging sharing economy (which incidentally have played similar roles in developments in non-industrial sectors such as logistics and health care). Nor will I delve into the issues of increasing vulnerability as a result of ever-more complex data acquisition and aggregation, although these are surely very important topics for employment-based societies and for businesses. This narrow focus intentionally replicates the narrow confines in which the discussion about Industrie 4.0 itself has taken place. Doing so makes it possible to analyze thoroughly and critically the discourse and the phenomena it addresses.

My observations begin at the purported discursive roots of Industrie 4.0—in the assumption that it arose in reaction to a new quality of technological development and that its intellectual home is above all in Germany, where the economy is still highly influenced by engineering and industry.

The “Industrie 4.0: Understanding a Seemingly Technical Debate“ section provides an introduction for readers with only cursory knowledge of Industrie 4.0 and provides the necessary background for the actual analysis, which begins in the “Future told: a Global Strategic Discourse“ section with a review of the origins, progression, intention, and actors of a discourse that has a lot more to do with economics than technology.

On the basis of this critical discourse analysis, and drawing on the theories of Barbara Adam’s and Chris Groves’ *Future Matters*, “Future told: a Global Strategic Discourse,“Future traded: the False Hope of a New Source of Unbridled Growth,“ and „Future tamed: the Global reorganisation of work“ sections elaborate the central argument of the paper that *Industrie 4.0* (or, rather, that which is referred to by this name in Germany) should not be interpreted as an example of “visioneering.” With respect to the German debate on Industrie 4.0, it is shown that German engineers are not the driving force behind the debate, nor are they interested in “building things” (as noted above). McCray’s visioneers use their visions to raise money and attract supporters and thereby achieve their technical goals. Their visions are a means to an end, or, as Nordmann puts it: “What visioneering aims for is to exhibit a compelling causal link between a state A (technological work-in-progress) and a state B (a future so desirable as to mandate its realization) (…)” [5].

The empirical basis for this article is a discourse analysis of a wide range of texts published between 2009 and 2015, which follows the “sociology of knowledge approach to discourse (SKAD)” [6]. More than 220 publications were chosen and, in line with the SKAD, were analyzed with regard to the explicit opinions they contained and the economic interests, social relations, and politics of knowledge that the future visions in this material addressed.

Following Adam and Groves, I argue in this article that Industrie 4.0 should be understood as a process that works in a manner contrary to the way in which it is often conceived. Industrie 4.0 is not a debate about “the embedded, embodied, contextual future” but a phenomenon of a “decontextualized future emptied of content, which is open to exploration and exploitation, calculation and control” ([1], p. 2).

The three main sections will discuss the debate on Industrie 4.0 as an intentional *future told* from Adam’s and Groves’ analytical perspective. The “Future told: a Global Strategic Discourse“ section elaborates on the manner in which agenda-building and big narratives have been deliberately initiated by influential actors on a global scale. The “Future traded: the False Hope of a New Source of Unbridled Growth“ section proceeds to show how Industrie 4.0 could be seen as a form of *traded future*: a future that only appears utopian to a superficial regard. In terms of certain well-known economic considerations that are highly pertinent today, this future fails to live up to its self-imposed utopian aspirations. The overall aim of Industrie 4.0 seems not to be the development of visioneering forms of trading that would be genuinely utopian and truly novel (in generating post-growth prosperity, for instance, or sustainably using global resources and promoting a more equal distribution of wealth) but to ensure the persistence of the current economic spatio-temporal logic. In order for this traded future to be established, production and distribution processes not only have to be digitized and integrated into a global network, but—as the „Future tamed: the Global reorganisation of work“ section shows—also need to be restructured from the global level down to every workplace and shop floor. Discussions of Industrie 4.0 and the vision of a Fourth Industrial Revolution therefore also tend to paint a picture of a *tamed future*. The “Industrie 4.0: a Future Told, Traded, and Tamed“ section brings together the key results of the discourse analysis. These results are contrasted with an interpretation of the German debate on Industrie 4.0 that at first glance appears accurate, namely, one that construes this debate as a nationally centralized and technologically driven case of visioneering. The article closes with some theoretical reflections on the significance of the sociomateriality of technology and the societal and economic role of human labor in realizing visions of the future.

## Industrie 4.0: Understanding a Seemingly Technical Debate

After being introduced to a wide audience for the first time during the Hannover Trade Fair in 2011, the Industrie 4.0 concept migrated quickly out of the specialized discourse to become a widely recognized catch word in the popular vernacular. It succeeded in putting industrial production, a dead topic for many years, back in the limelight. What had been written off as “old economy” became celebrated as the nucleus of an IT-based revolution. Industrie 4.0 was elevated to a central strategic goal for economic and industrial policy [7]. The evolving discourse around Industrie 4.0 became very diverse, complex, and interest-driven as the list of technical options associated with the label was lengthened almost daily. For the initiators of the debate, Industrie 4.0 is nothing less than the fourth industrial revolution: “The first three industrial revolutions came about as a result of mechanisation, electricity and IT. Now, the introduction of the Internet of Things and Services into the manufacturing environment is ushering in a fourth industrial revolution. In the future, businesses will establish global networks that incorporate their machinery, warehousing systems and production facilities in the shape of **Cyber-Physical Systems (CPS)**” ([4], p.5; emphasis in original).

The terms *Internet of Things* (IOT) and *cyber-physical systems* indicate what is at stake here. It is about networking digitally the maximum possible number of elements involved in production processes, support services, and logistics. In this way, the material melds with the digital. Everything from a local production line to global value chains is to be globally networked and locally regulated, as in the often-cited example of highly customizable products. All manipulable objects in the production process are to be fitted with data transponders that communicate position and production status to the machines doing the processing work. In “smart factories” built on these principles and in the “smart service welt” that emerges afterward [8], the restrictions of traditional mass production can be overcome. The idea is that products will be custom-manufactured in response to individual needs and only on demand.

At every point on such complex production and value chains, an immense amount of data is created, even today. In the course of CPS, the volume of data that has to be integrated will increase exponentially. Not only the elements being moved through the production process but also all of the sensors and activators of all the machines and plants involved generate status data continuously, and this data stream may even one day be extended to driverless systems that deliver the finished product right to the customer’s door. This is big data indeed, and not only data about human consumption or health behaviors but also data about machines and human-machine interaction such as the longevity of machine parts or optimal delivery routes. Note that all this is not simply about stretching an additional layer of data over already-existing databanks and applications. Other technical developments also play a central role in the Industrie 4.0 scenarios being discussed:

- To date, we are familiar only with large stationary or axial industrial robots. In the future, light robots, two-armed robots, and adaptive “robotics” will make new forms of human-machine interaction possible, making the use of robots feasible for tasks that cannot now be profitably automated.
- Completely new production technologies are also being considered. “Additive manufacturing processes” or 3D-printing makes decentralized, on-demand production of individual parts possible. Approaches such as “rapid tooling” change the way businesses make tools and thus also change the work process at the point of production.
- Finally, “wearables” also come into play. These are digital devices that can be affixed directly on workers and can include anything from data glasses that display information during maintenance work to the smart glove that sends a warning signal when a worker assembles something incorrectly or assesses assembly workers’ stress levels by monitoring their vital signs.
- This is by no means an exhaustive list of possible technical applications relevant for Industrie 4.0. These also include all of the technically supported capabilities so well known to most of us from our private use of smartphones, tablets, apps, and social media. All of these technical capabilities and more can be integrated into the factory workplace.

This quick review includes only a small part of the technological *tour de force* supposedly awaiting us in the future, and it makes little sense to elaborate further on the important technical details that underlie them. The sheer expanse of possibilities is overwhelming, but a healthy skepticism is called for because all of the speculation so far is in fact based on long-established technologies that are being improved incrementally. The often cited “teach-in” method of intuitive robot programming, for example, has been in industrial use for decades. Similarly, the use of data generated by machines and plants for remote or preventive maintenance is by no means new. Even the digitalized factory is virtually standard practice in mass production today. Large automobile manufactures, for example, give each chassis on an assembly line its own barcode. The barcode is affixed at what is appropriately called its “christening” in industry jargon before it moves on towards its “marriage” with an engine block. Incidentally, IT companies have been working for years (not yet with complete success) on methods to create an unbroken product data stream through all points of the product life cycle, from construction to disassembly.

Yet, some scenarios are being imagined that are genuinely futuristic. For example, 3D-printing, although nearly universally overhyped, is still in its infancy for many industrial uses. And whether the decentralized, self-directed manufacture of products in lots of one will ever prove to be economically sustainable and technically sufficient remains to be demonstrated. Making things additionally difficult is the fact that we have not even begun to resolve all the security and privacy issues that accompany these envisaged innovations. Nor is the needed technical infrastructure in place. Creating a sufficiently fast internet might well necessitate abandoning net neutrality, which would bring up political issues, and solving these would require negotiation among societal actors, not just a technical fix.

As the discussion above makes clear, there is no *single* Industrie 4.0. What innovations will be adapted in which branches and by which companies depends on the specific settings as defined by factors including but not limited to the degree of automation, product complexity, value chains, and production technology. We have little idea about these factors, even in today’s economy. Our current knowledge of the presence and influence of these factors in German industrial production is based on speculation. No official statistics are kept, for example, on how many people work at hybrid (half-automated, half-manual) assembly stations or which companies use anticipatory, data-supported maintenance. Given the lack of robust data, we must be very cautious with prognoses.

One can reasonably argue that the scenarios propagated in the Industrie 4.0 discourse may also be subject to *systematic* data bias. The tasks performed by industrial workers in advanced industrial societies are in fact highly diverse and complex, yet the available empirical labor market datasets that attempt to measure work tasks reproduce this complexity only crudely and superficially. This problem affects Carl Benedikt Frey and Michael A. Osborne’s often cited study [9] and German labor market research alike. The available surveys falsely assume that practically every human task on a machine is simple and routine and can thus be easily replaced by automation. This is more than a pragmatic simplification—it is a misleading assumption devoid of theoretical justification [10]. Moreover, simple *logical reasoning* also cautions us to be skeptical. If we take the discourse’s claim of immanent “revolutionary” and “disruptive” developments at face value, we actually have zero basis to make robust predictions of future events, as one can only predict the course of future events on the basis of previous experience. On top of this come plausible *economic* objections: innovations have never prevailed in industrial manufacturing simply because they were technically feasible. Were this otherwise, Germany would now be the home of a vibrant textile industry that had incrementally modernized its production technology for generations, given that textile manufacturing is the textbook case of the possibilities of extensive automation. This is, however, not the case. Apparently, it was more profitable for most German textile companies to use low-tech methods in low-wage countries rather than high-tech methods in high-wage Germany.

In sum, there are data-bias, logical, and economic reasons to be skeptical of any vision of the future derived from quantitative projections of labor market trends and based on a single-minded consideration of what innovations are now technically feasible. These kinds of Industrie 4.0 scenarios are short-sighted because they are not informed by qualitative insights into the reality of the working world and corporate strategy, e.g., ignoring the role of humans in human-robot interaction [11]. Nonetheless, it cannot be denied that these visions do have a certain degree of influence. Tomorrow’s reality need not be today’s reality; it can always be changed by powerful economic and political actors. The next section discusses these actors and their intentions.

## Future Told: a Global Strategic Discourse

The Industrie 4.0 discourse is often shortsightedly focused on technical issues and national boundaries, despite the fact that its origin and development were motivated more by economics than technology and influenced more by the international strategic environment than by national economic policies. The pervasive presence in 2015 of talk about Industrie 4.0 does not causally result from society having reached a new stage of technological development. It is first and foremost the result of professionally managed agenda setting. From the perspective of discourse analysis, this is a clear case of successful public relations.

The origin of the Industrie 4.0 discourse and all the ideas that were present at its creation were decisive in the formation of the discourse as it exists today because they vitally influenced the initial direction and content of prognostications about change. In Germany, the expectation that Industrie 4.0 will spur immense growth is closely linked to the strong export and innovation performance of German machine tool, plant equipment, and motor manufacturers. Not by accident do these companies play a starring role as providers of infrastructure in Industrie 4.0 scripts. Germany’s competitive advantages and the relatively high share of added value in industrial production favored a view of Industrie 4.0 that is filtered through the lens of national interest. The key actors very much want to see the global networking of manufacturing to be realized in such a way as to generate positive economic effects for the German economy. With a view toward global competition, especially with the USA and China, Industrie 4.0 has even been characterized as the question that will decide the fate of German industry [12]. Nonetheless, although the specific concept may have been a German invention, the idea that underlies it is not. An international debate that paralleled discussions occurring in (West) Germany is clearly evident.

Between 2009 and 2010, a number of widely noticed studies were published, mainly by business consultants. Written in the shadow of the international financial crisis, they fueled a rediscovery of the significance of the industrial sector despite previous long-term trends of deindustrialization. They expressed concern over the flagging competitiveness of established and once-strong industrial economies (Germany, Japan, USA) relative to the emerging industrial economies, especially those in Asia. The following messages played a prominent role in this discussion.

- An elaborate comparison of the complexity of national economies [13] ranked Germany first of 129 countries. Disappointingly for Germany, however, a later publication using the same dataset to estimate future economic *potential* placed Germany at the bottom of the ranking, near Yemen.
- The international corporate accounting and consulting firm Deloitte created the *Shift Index* [14]. This is a three-part strategy that conjoined, probably for the first time, many of the elements typical to the national Industrie 4.0 discourse. First, the strategy calls for the creation of decentralized steering mechanisms for new forms of collaboration and a “flow of knowledge.” Second, these are to be based on digital infrastructure and even greater market liberalization. Third, economic and political actors are advised to cooperate in the realization of these goals on so-called platforms [14].
- Germany was ranked 8th on the *Global Manufacturing Competitiveness Index* [15]. Although Germany remains technically advanced, the Chinese industry has clearly caught up and now even exceeds its German rivals in the area of wind and solar power technology. High labor costs and bureaucratic hurdles for start-ups were cited as demerits.
- Germany was ranked 13th on the *Enabling Trade Index* in 2010, good enough to make it the “best performer” among the large national economies. Germany placed well ahead of the USA, which had ranked 16th a year earlier but fell to 19th in the 2010 ranking [16].

Influenced by these studies and their strategic recommendations, three initiatives were launched during the World Economic Forum (WEF) in January 2011: a task force, the “Future of Manufacturing” project, and the Global Agenda Council on Advanced Manufacturing [17]. Participants in the new initiatives included corporate representatives from Volkswagen, Bosch, and Daimler. Among this group was also Siegfried Russwurm of Siemens AG, who later became one of the spokespersons of “Platform Industrie 4.0,” founded in 2013. These WEF organizations see themselves as a “platform for informed dialog between senior business leaders and policy-makers.” To kick off activities at the WEF level, they soon agreed to promote a “data-driven narrative” in support of “the strategic use of public policy as an enabler of economic development” [17], and 3 months later in April 2001, the term Industrie 4.0 was floated at the Hannover Trade Fair as noted above [18]. After this successful beginning, the discourse never quieted down. It even seeped into the popular vernacular. In the process, political actors at the European, national, and federal-state levels started to play in exemplary fashion the “enabling” role imagined for them by the WEF. In addition, all of the large corporate consulting firms have taken up the narrative as initiated and quantitatively undergirded by the WEF, contributing their own data and details. The plot forged in Switzerland bore fruit.

Ironically, the same consulting firms that joined the vanguard recommending intentional deindustrialization now point to the industrial sector not only as the core element of the value chain but also as the essential prerequisite for the preservation of “high-quality services” in the national or regional economy [19]. In a study of how to create an Industrie 4.0 ecosystem, Roland Berger Strategy Consultants offered up a crystal-clear plan centered around the promotion of Industrie 4.0 as a European idea and legislation to fund start-up friendly infrastructure [19]. These appeals are of course addressed to European and national-level politicians.

These initiatives have resonated greatly, as superbly exemplified by the Industrie 4.0 discourse in Germany. It can hardly be denied that political actors are carrying out their part of the plan dutifully. Note that this assertion requires no need to assume the influence of some kind of causal force “from above,” guiding the dynamics of national public discourses and ensuring their success. The conjunction of diverse personal alliances, the influence of corporate consultancies, and the economy-driven worldview shared for years by politicians and corporate leaders are fully sufficient to ensure that reality remains consistent with the master plan.

One thing, at least, is clear. Industrie 4.0 got its discursive wings not primarily from the rise of new technical possibilities but rather from economic “exigencies” as identified by economic elites. In the initiation of this elite discourse, German business leaders did not play the key role, although without a doubt Germany is a strategic and internationally valued power player, and the members of the “Plattform Industrie 4.0” are surely proud to have kept the German spelling of their group from being Anglicized. Looking back from today’s perspective, however, we see that Germany was neither the inventor nor the protagonist in a narrative that other actors brought into play with the intention of reinventing the world of industrial production.

## Future Traded: the False Hope of a New Source of Unbridled Growth

In elaborating their notion of a “traded future,” Adam and Groves distinguish between an *abstract*, an *open*, and an *empty* future. The abstract future, “belonging to everyone and no one,” inherently calls for—and contradicts—the concept of an open future, which is described as “belonging to some degree to human beings themselves; as produced through human intervention supported by an awareness of freedom and potentiality” ([1], p. 57). Since the concepts of the abstract future and the open future are entangled with each other and fraught with numerous tensions, they were increasingly replaced with what the authors call an “empty future”: the “central problem associated with the open future, i.e. which potential future to choose, is solved using mathematical methods to quantify the prospective gains and losses entailed by each of the alternatives.” Adam and Groves identify both a domain—“the appearance of an autonomous market economy towards the end of the 18th century”—and a set of willing actors—“an independent social science called economics” that jointly laid the “foundations (…) for the cultural dominance of the empty future” ([1], p. 57). Both the market economy and modern economic science subsequently acquired an ally that has helped bridge both spheres and enhance their mutual interaction: consulting firms are introduced here as the main drivers of the narrative, promising a future of everlasting growth as long as society willingly follows the path and pace they set down.

In the origin and course of the public debate on Industrie 4.0, we see that certain economic expectations attained priority. The focus was never on technical innovation, which was always treated merely as the means for securing greater economic growth. And indeed, the projections of the potential growth effects of Industrie 4.0 are very rosy. Some analysts have ventured the prognostication that Industrie 4.0 will bring economic growth for Germany in the amount of 78 billion euros by 2025 and a growth rate of up to 30% in some branches such as machine tools and plant construction [20]. The methods used to arrive at these projections, however, are questionable. Those 78 billion euros supposedly represent the isolated effect of Industrie 4.0, that is, its effect separate from growth due to other factors. The study picked out six especially innovative economic branches for analysis: chemicals, automobiles, machine tools and plant construction, electrical equipment, agribusiness and forestry, and information and communication technology.^1^ For all other branches and under the assumption of such growth rates, the study adds a 50% growth multiplier to arrive at the prediction that Industrie 4.0 will by itself spur economic growth in the amount of 267.5 billion euros for the German economy alone [20]. Upon closer examination of the study’s methods, it is clear that these are black-box prognoses, although admittedly this does not prevent the research unit of the Deutsche Bank from re-using them uncritically [22].

The numbers sound fantastically good and are happily accepted in the halls of politics. However, a recent systematic comparison of studies assessing the potential impact of Industrie 4.0 criticized their lack of methodological rigor and showed that while Industrie 4.0 would indeed be likely to spur economic growth, the expense of the necessary investments would most likely cancel out any overall growth effect [23]. Advocates apparently overlook something every entry-level manager knows: investment costs have to be subtracted from revenue in a correct profit calculation. But as long as economic growth is measured solely as gross domestic product, even poor investments count as growth. Nonetheless, the statistical legerdemain intended to make Industrie 4.0 shine is not intentional duplicity, it is just another example of a pervasive flaw in business school thinking referred to as “the capitalist’s dilemma.” In their article with this title, published in the *Harvard Business Manager* of all places [24], Christensen and van Bever take their own colleagues to task and argue against a promoting innovation at any cost. Instead, we must differentiate between “market-creating innovations” and “efficiency innovations.” Most Industrie 4.0 scenarios link growth projections to increased flexibility of production with supposed concomitant productivity gains. This, however, is just another form of efficiency innovation, unsustainable from the Christensen and van Bever perspective. Greater efficiency serves, they argue, only to eliminate jobs (or to prevent new jobs from being necessary) and to generate additional capital. Yet, corporations already have more capital than they want to invest. The authors estimate that corporations are currently sitting on more than 1.6 trillion dollars in unused capital. This money should be invested in market-creating innovations.

The worrisome state of the global environment alone would justify a worldwide negative growth program, but if the Industrie 4.0 vision becomes reality, there is no indication that any of its aspects would run counter to the dominant ideology of economic growth. It would only serve to heighten what a German sociologist calls the dual crisis of economy and ecology [25]. Sure, insofar as Industrie 4.0 means on-demand-only production, fewer natural resources would be wasted, but a powerful reason to doubt that Industrie 4.0 would ever be green can be found on the assembly lines of every major automobile manufacturer today. Even on these lines, designed for high-volume production, every automobile is individually identifiable from its “christening” onward and can thus be fitted at every point of assembly with individualized options. Thus, production in series even now is by no means to be equated with rolling out identical products en masse. The status quo of today’s data network and assembly line technologies has advanced enough already to allow companies to produce individual automobiles only after receiving a specific order from an identifiable customer. No doubt, the technology associated with Industrie 4.0 would improve the capabilities of such a production regime. All supply process could be integrated into self-directed, decentralized value chains optimized for the manufacture of products in lots of one such as to eliminate overproduction. Yet, as long as the logic of unbridled growth remains dominant—for example in the dogma of “overall equipment efficiency” (OEE) and its dictate to maximize the utilization of plant and equipment—the new capabilities of self-directed CPS will not be employed to improve environmental outcomes.

An additional consideration is the unheard-of growth that Industrie 4.0 is expected to spur through the integration of the Internet of things. A prognosis ventured by the corporate consultancy McKinsey arrived at the figure of over 11 trillion dollars per year (!) in additional growth through 2025 [26]. However, this calculation assumes a price for rare elements, essential for many IoT components, that is already much too low from an ecological perspective, and the study further assumes that the prices for other essential components such as micro-electromechanical sensors will sink by 30 to 70% [26]. Imagine, however, the consequences of such a dramatic fall in prices in terms of the tide of global electronic garbage, which grew globally by 42 million tons in 2014 alone.

Industrie 4.0 and the Internet of Things certainly are feasible technologies and could make a welcome contribution in the near future to the reduction of resource consumption. Yet, they are hardly likely to break the dogma of growth and will instead only greatly exacerbate the dual crisis of economy and ecology.

A supposedly dangerous threat to economic growth remains one of the main diagnoses undergirding the WEF’s “advanced manufacturing” strategy [17], the most serious problem being the US economy. The “Big Shift Index,” put out by Deloitte in 2009 for the first time, showed that return of assets (ROA) in the USA has been in continual decline for the past four decades despite some work productivity gains [14]. The data were positive only for the highly subsidized aeronautics and defense industries and for consumer goods production. This noteworthy finding could be interpreted as empirical support for Marx’s hypothesis of the inherent tendency of profit rates to fall as a consequence of increasing technical automation. This leads to a reduction of the significance of human labor for value creation and is for Marx one of the central mechanisms pushing capitalism to its inevitable end. Deloitte would not concur. They explain the cause of falling profits differently:

…[C]reative talent continues to capture increasingly disproportionate returns in terms of total compensation relative to the rest of the labor force…. On the customer side, new generations entering the marketplace appear to be more willing to exercise their market power to switch to products and services…. *The growing power of creative talent and customers … helps to resolve the mystery of why ROA is declining so markedly at the same time that productivity improvements continue to occur* ([14], p. 12; emphasis in original).

This explanation is surprising because it ignores standard explanations for the fall of profitability, including for example increased global competition, disruptions of financial markets, the most recent crisis, and mismanagement. Instead, Deloitte argues (without providing empirical evidence) that the cause of the problem lies in the influence of workers in the creative sectors and in consumers’ purchasing power. The first group, which stands at the upper echelon of the qualification pyramid, earn too much, even as consumers prefer cheaper services and goods. Apparently, the argument is that companies in innovative markets must pay their workers high wages without being able to pass these costs on to the consumer because of stiff price competition.

For Deloitte, the right response is not “to squeeze creative talent and customers in a zero sum battle to capture more of the existing pie [but rather] to discover new ways of organizing and operating to more effectively create and capture new value” (ibid.). The consultants do not target disproportionally high salaries of creative workers for change. Rather, they recommend that corporate managers organize new and efficient ways of valorizing human work. From this perspective, then, human work in the spheres of production and consumption has gained significance for the promotion of growth and the generation of profits.

## Future Tamed: the Global Reorganization of Work

For decades, jobs have been replaced by automated systems in a continual process of labor rationalization. In the 1980s, armies of typesetters and analog print workers were swept aside by new printing technologies. And who today remembers shop-floor secretaries? Or the fact that “bank teller” once used to be a synonym for secure employment? Or the days when more humans than robots worked on Germany’s automotive assembly lines? These and other profound changes in work organization came about relatively unnoticed, and the topic of technology-induced unemployment has returned now to the public agenda only because of new, Industrie 4.0 production technologies. These technologies are, with good reason, thought to have the potential to completely transform productive labor as we know it. Intelligent algorithms and Big Data could replace qualified knowledge workers, at least partially, and inexpensive light robots are likely to be used in production environments that have so far resisted automation. And incidentally, if the driverless car ever becomes a practical reality, it will dramatically change all economic activities dependent on package delivery and small-scale transportation.

Furthermore, Adam and Groves show how and why futures have to be not just told but also *tamed* ([1], 39–56). In predicting the future, and a fortiori in creating it, human beings have to cope with its underlying pitfalls, particularly those of uncertainty and mortality. While ancient societies were able to embed this endeavor in rituals and/or delegate it to divine authorities, modern societies—whose complexities and enhanced velocities of change produce greater uncertainty on a potentially larger existential scale—cannot rely on ancient modes of coping, but require different ways of taming the possible downsides of the future.

At an individual level, grave uncertainties and mortality serve to reduce life to the level of mere existence, while at societal and historical levels, the accumulation of risks serves to endanger humanity as a whole. Between these existential dimensions of mortality, in the sphere of the economy and labor, existence and non-existence are translated into doing business and the dying of business on the one hand, and into having or not having a job on the other. As a *future told*, Industrie 4.0 also needs to *tame* the uncertainties and risks posed by “mortality” for business and jobs.

In light of the potential magnitude of the predicted economic and societal changes, then, it is no surprise that a large number of studies have addressed the manner in which automation has the effect of eliminating jobs, where “job elimination” may well mean the elimination of *all* jobs in any given production environment through the replacement of human workers with intelligent things [9, 27–29]. As noted above in the “Industrie 4.0: understanding a seemingly technical debate“ section, in reflecting on the potential large-scale transformation of labor, the fundamental methodological problems inherent too much of the Industrie 4.0 debate should not be downplayed. Nor should we neglect the fact, also noted above, that current prognoses about the possible effects of new technology on employment are often diametrically opposed to one another. Note the contradictions for example in debates on workforce re-qualification versus workforce dequalification, or on whether automation liberates workers from repetitive tasks so that they can be more creative or rather makes them wholly redundant.

Putting aside these objections for the moment, however, it is also clear that to focus narrowly on individual technical options, on current methods of effecting a division of labor, or on the kinds of job tasks now in use misses the main point completely. And that is how the visions of Industrie 4.0 are taming the risks and uncertainty of business right down to the shop floor. Again, without ancient gods to turn to, when modern societies envision the Fourth Industrial Revolution, they rely on something they invented during the first one and have been optimizing ever since: the (seemingly) perfect plan—a plan that promises both: to anticipate possible uncertainties and to provide tools to cope with any minor ones that may be left over. Today, these tools comprise all forms of organizational standardization [30]. With Industrie 4.0, these concepts of planning and standards have to be propelled into a globally connected economy and have to be transformed to fit in, as we see now as we return to the discourse analysis on Industrie 4.0.

Current visions of a future with globally networked economic actors re-conceptualize the working world on a much, much larger scale, placing the management of all global value chains at the front and center of discussion. The goal is to create structures free from local connections, regional expertise, and labor market-specific configurations. This is about creating *globally standardized, networked production and service structures that enable the flexible and self-directed collaboration of fixed and variable capital*. One might discount this project as a utopian dream of capitalist visionaries with no basis in reality. Yet, a careful examination of the discourse as reconstructed in the sections above makes clear that the projects being discussed contain specific goals that are even now close to implementation. Due to space limitations, we offer below only one—highly relevant—piece of evidence to support this argument: the WEF’s use of the old Copy EXACTLY strategy as a case in point, illustrating the feasibility of its recommendations.

At first glance, it is confusing that the WEF uses a strategy called “Copy EXACTLY!” pioneered long ago in the 1980s, as a case in point to illustrate its vision of the future [31]. Why indeed did the WEF choose to revisit this old strategy, initially developed by Intel? [32].^2^ It is worth studying the original project at length because it provides insight into the mentality of the Intel managers who invented and implemented it. Their core idea, addressed to the problem of how to best expand manufacturing capacity globally, was to build multiple exact copies of running manufacturing lines on different sites around the world. “Stated in its simplest form, everything which might affect the process, or how it is to run, is to be copied down to the finest detail, unless it is… *physically impossible* to do so…” ([32], p. 2). As it turned out, virtually nothing *physical* proved impossible to replicate—right down to the smallest details such as the color of assembly workers’ gloves. The problems that Intel encountered trying to set up an exact replica of an American factory on European sites were *immaterial*: “Making a philosophical statement is obviously much easier than implementing it within a large team of R&D and manufacturing engineers” [32]. Intel’s European technicians and engineers, headstrong and better trained than their American colleagues, were irritated by the directive to merely copy an American production line. This offended their professional ethos, which places a high value on continual improvement. An analysis of the project concluded by citing an astounded American Intel manager’s observation that European educational systems train graduates to think independently, which meant that the order to copy felt to them like an order to “cheat” ([32], p. 3). This impasse spurred a corporate learning process. For neutralizing opposition to their strategy, based on the professional identities of their European employers, Intel eventually came up with a four-stage implementation system that made concessions to local creativity but still created assembly lines that exactly copied American production lines.

What is noteworthy about Intel’s implementation process was that the regionally rooted skills of the headstrong European engineer were neither played down in their significance nor technically substituted. The solution was to shut down those aspects of engineering knowledge that are regional and unique only temporarily during the copying phase. Later, when the line is up and running, the copying phase ends, and the regional expertise of the European engineer is re-activated and indeed may be massively rewarded. Ideas for improvement that have proven effective in one plant are implemented in all similar facilities worldwide (these had to be implemented within 1 week!). Thus, identical production facilities worldwide means not only that the benefits of engineering know-how are no longer limited to local plants but also that the total sum of actionable production improvements worldwide increases without increasing the number of technicians overall. “In effect, the number of engineers per process step or per area for improvement is increased, as is the number of improvement ideas generated“ ([32], p. 5). The system extracts the innovative capacity of local engineers from specific regional environments, thus increasing their effectiveness, while at the same time guaranteeing an especially high flexibility in production: “With three sites running the exact same process, products were easily transferred back and forth with no re-qualification. Using free capacity at another site has also solved manufacturing bottlenecks” (ibid.).

Although the “copy EXACTLY!” method started out simply as one company’s strategy for optimizing trans local production methods, for the WEF it is an example and instructive blueprint for complementing more recent technological advances. The underlying goal for all is to optimize process efficiency by making production less dependent on locally oriented and situation-specific knowledge—as when, for example, a medium-sized company outsources an algorithm-based optimization of its “milk runs” (the intra-logistical routes by which parts are delivered to machines on the factory floor) to a service contractor’s data server, or when maintenance-relevant data from countless sensors and activators in a complex production facility are analyzed using big data in order to prevent malfunction, or when a technology like 3D-printing is used to shorten the time it takes to go from prototype to series. All these are examples of a completely new, flexible, and globally manageable division of labor, and as it becomes extended by techniques such as crowd sourcing or crowd working and is applied in different market contexts such as “sharing” or “platform” economies, new and highly variable constellations will emerge that combine value creation and value realization, off-line and online, and commodity and commons in completely new ways [33]. Of course, this kind of globally networked mode of flexible production requires local correspondence in the specificities of work organization, at least in respect to human-machine cooperation. Humans and machines should be as self-directing as possible but should most especially be able to adapt to continually changing conditions of production. The demands this model places on workers’ willingness to collaborate are high, and thus, it is not surprising that the discussion of the democratic, participatory firm is heating up again. Within this discussion, the interaction between humans and machine is of particular relevance for Industrie 4.0.

It is being said that knowledge—and with it, human work—is now important again. However, the forms of useful knowledge and thus of human work are changing very rapidly. Many agree that rapidly developing digital infrastructure and years of deregulatory politics combine to create a second-order effect: “They unleash a flood of knowledge flows on a global scale that become more diverse and richer with each passing year.” In this context, the knowledge corporations need is in constant flux. Firms cannot afford to merely conserve already-acquired knowledge but rather need to adapt a strategy of permanent knowledge acquisition: “Now, there is an opportunity to learn faster and drive more rapid performance improvement than ever before by harnessing these knowledge flows” ([34], p. 28).

To cite a second voice in the ongoing discussion, the Bosch manager Siegfried Dais echoes the views of many others when he claims that we are on the cusp of an “elemental paradigm shift” quite unlike the CIM ideas of the past [35]. Dais advocates replacing older production models based on central planning and robust but rigid value chains with models based on decentralized self-management and the ad hoc organization of value-creating networks ([35], p. 613). In his estimation, we need to create an “architecture and rules for a value-creating network that links millions of decision points globally, is secure and robust, and is characterized by high availability” ([35], p. 633).

At one point, Dais asseverates: “the pace of production will be a human pace.” Yet, looking again at his entire argument makes one rather skeptical about this. If the employment of human labor is described in the same breath as both highly flexible and dependent on expertise needed *in a certain span of time* ([35], p. 614), the author appears to be just paying lip service to the abilities of individuals to influence the pace of production. Note, for example, that the German logistic branch has sent out signals that logistics 4.0 is only to be had if changes in production sites can be undertaken with quicker response times in the future. “The whole thing begins with the fact that the best location for a system can no longer be determined on a long-term basis due to the ever more volatile production and trade environments. There is no such thing as the ideal production location, sometimes for many years. The logistics network and its nodes must be continuously adjusted to circumstances. Thus, the logistics nodes of the future will have to be moveable” ([36], p. 615). Humans and intelligent machines increasingly melt into a “blended workforce” in which technology is not just a set of physical tools but is rather the “newest employee” and should be seen as one, indeed as a “partner in a new collaborative workforce“ ([37], pp. 88–89). The WEF uses nearly identical definitions, also favoring the term blended workforce, and characterizes “digital labor” in such a way as to make no distinction between variable and fixed capital, between (wo)man and machine. “Digital labor” is a specific reference to the use of digital technologies to take over work once done by humans: “...smart sensors, machines (e.g., robots), or intelligent systems that can do parts of the jobs that only humans used to do” ([38], p. 28).

What remains puzzling in both visions is just how all this is going to make human work more autonomous and decentralized as its proponents repeatedly claim. If humans, machines, and intelligent systems are to be transformed into a globally networked, digital-human blended workforce ([38], p. 7) in which the individual components are universally deployable in a highly efficient, self-directed collaborative production system, how will its human components be free to concentrate on the “more human elements of their jobs like creative problem-solving and collaboration” ([38], p.17)?

## Industrie 4.0: a Future Told, Traded, and Tamed

The immediate effects of Industrie 4.0 on the industrial sector will be significant and far-reaching. Under no circumstances, however, should we lose sight of the fact that Industrie 4.0 is at best just *one* phenomenon among a wide range of disruptive global transformations. As mechanisms of corporate control increasingly prove unable to solve the basic problem of how to transform purchased labor power into actually rendered work, many prognoses and visions of the future organization of work tell us that this problem will or ought to be resolved through the self-direction of variable capital. It bodes ill, however, that even as these prognoses have spurred a renewed celebration of the “democratic firm” and “participatory decision-making,” our society is perfecting the means of collecting massive amounts of data on all aspects of individuals’ work-related activities, both as employees and as consumers. Is this digitally augmented hollowing-out of both the private sphere and of labor rights and the democratic potential of industrial relations not a new form of despotism? Here, we might observe that it is the key actors themselves who interpret their Industrie 4.0 plan to link global economics to new forms of production as a global, long-term strategy. Let us give the experts from Deloitte the last word, again with a quote from the 2012 WEF paper:

“To understand the future of manufacturing, we need to explore a much broader set of dynamics that are reshaping the global business economy. These powerful forces have been playing out for decades and will continue to unfold over many decades ahead (…). We call these forces and the trends they set in motion the ‘Big Shift’ ([14], p. 28).

As should be clear by now, Industrie 4.0 is a central element of this Big Shift. Building on Adam’s and Groves’ theoretical framework and drawing empirically on a discourse that stretches back to 2009 and includes publications from major actors in the global economy, this article began by debunking the myth of Industrie 4.0 as a vision formulated by German engineers. At first glance, the discourse of Industrie 4.0 might be interpreted as a case of *visioneering*—one that “connects this emphasis on design, engineering, and construction to a more distant time horizon and an expansive view of a future determined by technology” ([2], p. 11). McCray’s perspective proved to be an inspiring one, but other aspects of his concept of visioneering were more difficult to observe in practice: the discourse analysis did not associate technical engineers with brilliant and concrete technical ideas, nor did it identify them as individuals who use and potentially exploit a vision of the future to attract venture capital and entice supporters. The article rather analyzed the discourse on Industrie 4.0 as an example of agenda building, showing which global actors are shaping the narrative that has made the vision of Industrie 4.0 so effective and helped it to dominate public debate in Germany. Following Adam and Groves, we showed that the vision is embedded in a triple case of future-making: we first considered the dimension of a *future told* and traced the global strategic discourse that describes a new form of globally connected and almost autonomously functioning production. Secondly, we examined the underlying idea of a *future traded*, along with the perhaps misplaced hope for a new source of unbridled growth that this technological vision is predicted to unleash. And thirdly, we followed this narrative towards a complementary notion of a *future tamed*: a global reorganization of work that rewrites the role of human labor.

Adam and Groves make a key distinction between the “embedded, embodied, contextual future from contemporary perspectives”—a future that, as we saw in the “Industrie 4.0: Understanding a Seemingly Technical Debate“ and “Future Told: a Global Strategic Discourse“ sections, is to some extent still contained in the *future told* of Industrie 4.0—and a “decontextualized future emptied of content” ([2], p. 2). The latter is described as “open to exploration and exploitation” (in a manner we considered in the „Future Traded: the False Hope of a New Source of Unbridled Growth“ section under the heading *future traded*) and open to “calculation and control,” as detailed in the „Future tamed: the global reorganisation of work“ section. Our analysis drew on Adam’s and Groves’ understanding of “how the *emptying* of the future is implicated in both the progress of industrial-capitalist societies and the major problems that they face today” ([2], p. 2). They describe the latter as open to exploration and exploitation—we followed that path in the „Future Traded: the False Hope of a New Source of Unbridled Growth“ section—and open to calculation and control, as shown in the „Future tamed: the global reorganisation of work“ section. Adam and Groves inspire us with the idea of how the „*emptying* of the future is implicated in both the progress of industrial-capitalist societies and the major problems that they face today” ([2], p. 2).

Our sociological analysis was able to offer a number of insights into the nature, intentions, and dynamics of contemporary visions of the future by finely tracing the discourse patterns that create and transport these visions through different societal spheres. But if sociological analysis stops at the level of discourse, its perspective tends to become part of what Adam and Groves call the emptying of the future. Sociology cannot fill the substantial emptiness of a discourse by dissecting that discourse. But sociology can confront the emptiness of discourse about the future with its opposite: the embeddedness and thus materiality of work and production *today*. Whatever future form of production will become reality, it will not unfold in discourse alone; it will take place—or not—on the shop floor and be created and put to work by real people and their *living laboring capacity* [39], within real labor relations, using and creating real technology in all its *sociomateriality* [40].

## Footnotes

1 McKinsey is not as optimistic in their assessment of the growth potential in industrial equipment production. Unlike its predecessors, the fourth industrial revolution is not expected to be characterized by the wholesale replacement of the physical means of production. Rather, it will proceed more similarly to electrification. Many plants and machines will simply be enhanced with sensors, networks, and big data. Less than half are expected to be completely replaced, and the turnover is expected to occur over a longer timespan [21].

2 The “Copy EXACTLY!” orthography, chosen by Intel, was supposed to give expression to an intended, underlying “paradigm shift.”
